# AIRPred: A Deep Learning Model Predictor for Peptide Intensity Ratios in Cross-Linking Mass Spectrometry Improves Cross-Link Spectrum Matching

**DOI:** 10.1021/acs.analchem.5c03597

**Published:** 2025-08-27

**Authors:** Zehong Zhang, Mei Wu, Max Ruwolt, Ying Zhu, Pin-Lian Jiang, Diogo Borges Lima, Fan Liu

**Affiliations:** † Leibniz-Forschungsinstitut für Molekulare Pharmakologie (FMP), Berlin 13125, Germany; ‡ Charité Universitätsmedizin, Berlin 10117, Germany; § Freie Universität Berlin, Berlin 14195, Germany

## Abstract

Cross-linking mass spectrometry (XL-MS) is a powerful tool in structural proteomics, offering insights into protein conformations, interactions and dynamics by linking spatially proximal residues. However, current cross-linked spectrum match (CSM) scoring methods rely heavily on mass-to-charge ratio (*m*/*z*) comparisons, often neglecting fragment ion intensity information, which limits their ability to accurately distinguish true CSMs from false positives. To overcome this limitation, we present AIRPred, a deep learning model that predicts intensity ratios between cross-linked peptide pairs to improve CSM identification. AIRPred employs convolutional neural network (CNN) blocks to capture peptide fragmentation patterns and an attention layer to model peptide interactions. Our results show that intensity ratios remain consistent across experiments and can reliably differentiate true CSMs from random mismatches. In external validation, AIRPred outperformed traditional methods, demonstrating high accuracy in predicting intensity ratios. This model significantly enhances XL-MS analysis by leveraging intensity data for more accurate peptide identification.

## Graphical abstract

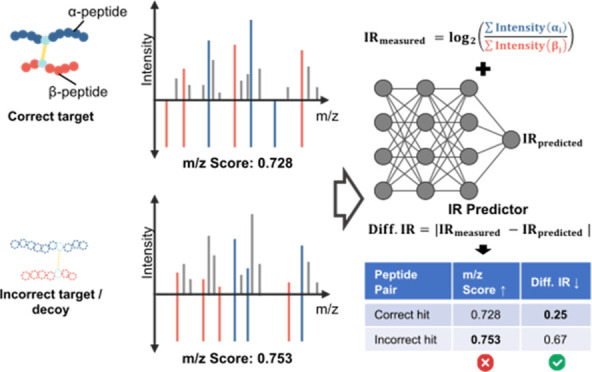

## Introduction

Cross-linking mass spectrometry (XL-MS) is a versatile method in structural systems biology, enabling the capture of large-scale protein interaction networks, binding interfaces, localizations, and structural dynamics by covalently linking spatially proximal residues.^1,2^ XL-MS can provide these insights directly from highly complex samples, such as intact human cells. Unlike traditional mass spectrometry approaches, which focus on single peptides, XL-MS involves identifying two peptides (α-peptide and β-peptide) connected by a chemical cross-linker, adding further complexity to the analysis. Particularly when analyzing native biological samples, XL-MS often relies on MS-cleavable cross-linkers such as disuccinimidyl sulfoxide (DSSO).^3^ Cross-linked proteins are digested with proteases (e.g., trypsin), typically followed by LC–MS/MS analysis using data-dependent acquisition (DDA) to identify the linked residues. This information provides valuable distance constraints, aiding the elucidation of protein conformations, topologies, and interactions. These constraints are particularly useful for understanding flexible protein regions, which often play important roles in protein conformational dynamics and are prime sites for drug-based intervention.^4,5^

Reliable cross-link identification requires scoring methods to confidently match candidate cross-linked peptides from the search database to experimental MS/MS spectra. Currently, most cross-linked spectrum match (CSM) scoring methods rely primarily on comparing the *m*/*z* values between theoretical and measured spectra.^6–9^ These methods optimize the scoring by ensuring that the identified fragment ion masses and types of the cross-linked peptides are consistent with the expected fragmentation patterns. However, many of these scoring processes do not fully utilize the signal intensities of the peptide fragment ions. Some algorithms like Comet^10,11^ make limited use of intensity information for weighting in fast cross-correlation calculations, but this approach lacks the depth needed to leverage intensity data to differentiate correct CSMs from false positives. Additionally, pLink2 incorporates intensity information as a feature in their reranking support vector machine (SVM) model.^12^ However, pLink2 only uses matched intensity rate (i.e., summed fragment intensity of both linked peptides/total intensity of the spectrum) as an additional evaluation of spectra quality. Similarly, MeroX allows users to filter spectra based on a minimum threshold of the summed fragment ion intensity of both linked peptides relative to the total intensity of the spectrum,^13^ serving as a quality control mechanism. Improving CSM accuracy requires more comprehensive scoring approaches supported by additional spectral features, such as fragment ion intensities.

Here, we discover that, for the same cross-linked peptide pair, the ratio of the summed-up fragment ion intensities of the two linked peptides remains constant across all CSMs. Accurately predicting this intensity ratio can help distinguish correct from incorrect CSMs (Figure 1). The relationship between the intensity ratio and peptide pairs, however, is complex and cannot be easily captured. To address this, we leverage artificial intelligence techniques, which have shown great promise in mass spectrometry applications, particularly in improving peptide identification and quantification.^14,15^ Our model, **AIRPred** (artificial intelligence intensity ratio predictor), employs convolutional neural network (CNN) blocks to capture peptide fragmentation patterns, along with an attention layer to model interactions between peptide pairs. This architecture harnesses the power of deep learning in XL-MS and provides a significant improvement over other baseline machine learning methods. **AIRPred** achieves high accuracy in predicting the intensity ratios of cross-link peptide pairs. In an external test set, using only the predicted intensity ratios, over 80% of true target CSMs were successfully distinguished from random mismatches. Additionally, through Shapley explanation analysis, we demonstrate that **AIRPred** not only predicted intensity ratio accurately but also provided molecular insights into the impact of amino acid properties on intensity ratios.

**Figure 1:**
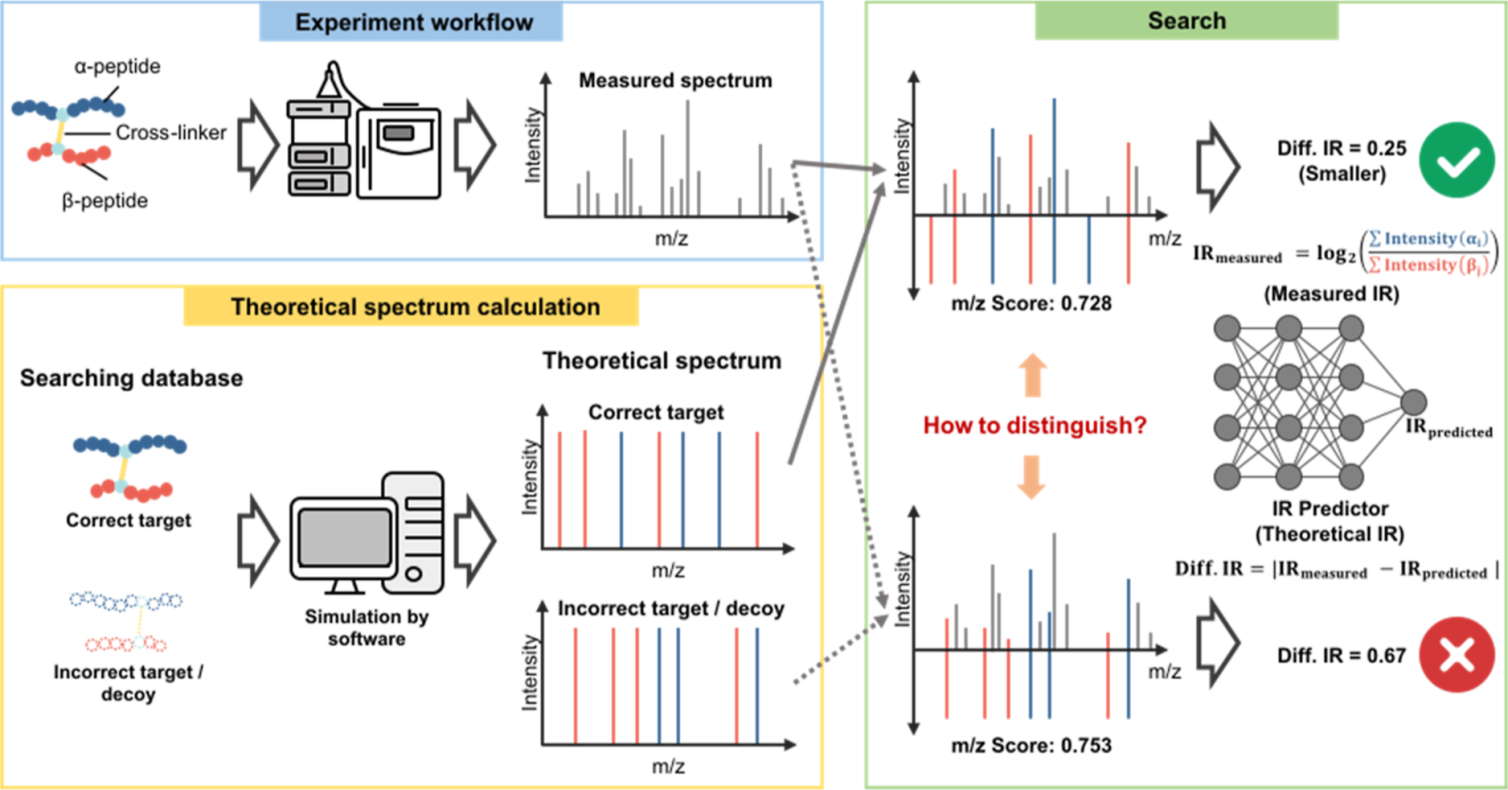
Limitations of traditional search methods. Sometimes confusion between correct and incorrect hits based solely on *m*/*z* Information.

## Materials and Methods

### Computational Environment and Software

All models were implemented using Python and trained on an NVIDIA GeForce RTX 4090 GPU and an Intel­(R) Core­(TM) i9-14900K CPU. The primary machine learning libraries used were PyTorch, Scikit-learn, XGBoost, and LightGBM. Data visualization and analysis were conducted with Python version 3.11.5, utilizing the following packages: NumPy, pandas, SciPy, Biopython, Matplotlib, and Seaborn.

### Data Processing and Data set Splitting

Mass spectrometry (MS) raw files were processed using Scout (version 1.5.0) software.^9^ Scout was employed to generate matching tables and calculate intensity ratios.

The ground-truth data set from Clasen et al.,^9^ generated using disuccinimidyl sulfoxide (DSSO) cross-linker, was selected for use as both the training and validation set. For cases where different scans identified the same cross-linked spectrum matches (CSMs), the average intensity ratio across scans was calculated and labeled as the ground truth. The total number of CSMs used was 20,961. To mitigate the impact of low-quality data on the intensity ratio, only CSMs with more than three occurrences were included in the training and validation sets.

For testing, we used a two-interactome (DSSO cross-linked *Escherichia coli* and HEK293T cells) data set,^16^ consisting of 975 CSMs. This test set was also generated using DSSO but differs from the training set in terms of species and MS parameters such as the higher-energy collisional dissociation (HCD) percentage (see Table 1). To evaluate the generalizability of the model to other cross-linkers, we employed a HEK293T data set,^9^ which uses disuccinimidyl bissulfoxide (DSBSO) as the cross-linker. Other MS parameters are similar to those in the ground-truth data set, making it suitable for assessing the model’s transfer learning capabilities. Additionally, three independent biological replicates of a mitochondrial XL-MS data set^17^ were used for data analysis. Due to the distinct fragmentation type from the training set (i.e., collision-induced dissociation), this data set was only used for analytical purposes and not for model training.

**Table 1:** Data Set Information

ID	data set	instrument	species	Frag@NCE^a^ (%)	no. spectra^b^	purpose (shown figure)	pride ID
1	ground-truth data set (first batch)^9^	Orbitrap Fusion Lumos	Homo sapiens (human)	stepHCD21-27-33	Total CSM counts: 20,961. Cross-link counts containing more than 3 CSMs: 8625. Only cross-links containing more than 3 CSMs are used in the analysis	train/validation/data analyzing (Figures 2A,C,D; 3A; 4A)	PXD042173
2	ground-truth data set (third batch)^9^	Orbitrap Fusion Lumos	H. sapiens (human)	stepHCD21-27-33		train/validation (Figure 4A)	
3	ground-truth data set (forth batch)^9^	Orbitrap Fusion Lumos	H. sapiens (human)	stepHCD21-27-33		train/validation (Figure 4A)	
4	two-interactome data set^16^	Orbitrap Fusion Lumos	H. sapiens (human); E. coli	HCD@21, HCD@24, HCD@27, HCD@30	total CSM counts: 975. All CSMs are used in the analysis	test/data analyzing (Figures 2D; 4B–D; 5A–D)	PXD031114
5	mitochondria data set (replicate 1)^17^	Orbitrap Fusion Lumos	H. sapiens (human)	CID@25	-	data analyzing (Figure 2B,C)	PXD032132
6	mitochondria data set (replicate 2)^17^	Orbitrap Fusion Lumos	H. sapiens (human)	CID@25	-	data analyzing (Figure 2B,C)	
7	mitochondria data set (replicate 3)^17^	Orbitrap Fusion Lumos	H. sapiens (human)	CID@25	-	data analyzing (Figure 2B,C)	
8	HEK293T data set^9^	Orbitrap Fusion Lumos	H. sapiens (human)	stepHCD19-25-30	308,810 CMSs (target: 189,346; decoy: 119,464). both target and decoy CSMs are used in the analysis	test (Figure 4F,G)	PXD043531

a Frag@NCE: Frag is the fragmentation type; NCE is the normalized collisional energy.

b Here, No. Spectra is the number of spectra used in training or testing of this model, or used to analyze intensity ratio’s property, identified by Scout.

### Baseline Models and Model Selection

A comprehensive description of the baseline machine learning methodologies used in this study is provided in the Supporting Information Methods. Briefly, we used the following baseline machine learning and deep learning models: support vector machine (SVM), random forest (RF), light gradient boosting machine (LightGBM), extreme gradient boosting (XGBoost), artificial neural network (ANN), and transformer. These models were used to establish performance benchmarks and guide the refinement process for the final model.

For model selection and optimization, we applied a combination of 5-fold cross-validation and random search for hyperparameter tuning. During cross-validation, the training data set was randomly divided into five equal parts. In each iteration, one part was used as a validation set, while the remaining four parts were used for training. This approach helped to comprehensively evaluate model performance and avoid overfitting.

Random search was employed to explore a wide range of hyperparameters for each model. Through this process, the baseline models were compared and refined to identify the best-performing model for the task at hand.

### Intensity Ratio

The intensity ratio *I*_α/β_ represents the log_2_ value of the intensity ratio between the α peptide and β peptide. Unless otherwise specified, all intensity ratios in this article refer to values processed using log_2_ transformation.

In this context, α_all_ denotes all possible fragments of the α peptide, while β_all_ denotes all possible fragments of the β peptide. The term *I* refers to the intensity of the matched fragments. α_*i*_ represents a fragment from the α peptide, where *i* ∈ *M* and *M* is the total number of possible fragments of the α peptide. Similarly, β_*j*_ refers to a fragment from the β peptide, where *j* ∈ *N* and *N* is the total number of possible fragments of the β peptide.

The intensity ratio is calculated using the following equations

1 $$I_{\alpha /\beta }=\log_{2}\left(\frac{I\left(\alpha_{\text{all}}\right)}{I\left(\beta_{\text{all}}\right)}\right)$$

2 $$I\left(\alpha \right)=\sum_{i=1}^{M}I\left(\alpha_{i}\right)$$

3 $$I\left(\beta \right)=\sum_{j=1}^{N}I\left(\beta_{j}\right)$$

4 $$I_{\alpha /\beta }=\log_{2}\left(\frac{\sum_{i=1}^{N}I\left(\alpha_{i}\right)}{\sum_{j=1}^{M}I\left(\beta_{j}\right)}\right)$$

### Mixed Score

DiffIRScore quantifies the discrepancy between the experimentally measured intensity ratio and the predicted intensity ratio from the model. Here, *I*_α/β_ denotes the measured intensity value from actual experiments, while *I*_α/β_′ represents the predicted value from our model. To prevent division by zero and limit score magnitude, a constant ε = 0.5 is added to the denominator. The DiffIRScore is calculated as the reciprocal of the absolute difference between these two values, as expressed in the following equation

5 $$DiffIRScore=\frac{1}{\left|I_{\alpha /\beta }-I_{\alpha /\beta }^{\prime }\right|+\varepsilon }$$

DDPScore (decoupled dot product score) is a spectral similarity metric used in Scout.^9^ It is an advanced spectral similarity metric that builds upon the spectral angle by comparing theoretical and experimental spectra through normalized vector dot products after binning. The key improvement is the exclusion of shared fragments between α and β peptides during binning to avoid bias. The formula is as follows

6 $$DDPScore=\sum_{i=1}^{M}\left(T_{i}\cdot E_{i}\right)+\sum_{j=1}^{N}\left(T_{j}\cdot E_{j}\right)$$

where *T*_*i*_ and *E*_*i*_ are the normalized intensity values of the binned theoretical and experimental spectra for the α peptide, respectively, while *T*_*j*_ and *E*_*j*_ correspond to the β peptide. Shared fragments are omitted from the calculation.

Mixed score combines the two scores using a weighted sum to evaluate the improvement gained by incorporating DiffIRScore into the traditional DDPScore. The weight parameter γ is manually determined based on the training set and was set to 0.02 in our study

7 Mixedscore=DDPScore+γ×DiffIRScore

### Model Interpretation

In XL-MS, CSMs involve two peptides linked by a cross-linker and *I*_α/β_ is influenced by both peptides. To isolate the effect of the α-peptide on this ratio, we fixed the β-peptide sequence and its modifications and utilized an interpretation method to assess the impact of α-peptide’s amino acid sequence on the intensity ratio.

For each peptide pair and its predicted intensity ratio, we calculated SHAP (SHapley Additive exPlanations) values for each amino acid in the α-peptide.^18,19^ These values quantify each amino acid’s influence on α-peptide’s intensity, with their total constrains to match to the predicted intensity ratio. This method integrates multiple approaches for feature attribution in prediction explanations.^20–24^

We employed DeepExplainer from the official SHAP implementation to interpret our model’s predictions (https://github.com/slundberg/shap). This tool estimates feature importance by comparing model outputs for specific feature values against reference values. To define these reference values, we generated 100 mutations of α-peptide pairs, replacing one-third of the amino acids with random ones. This ensures our analysis only focuses on amino acid effects on intensity while controlling peptide length variations.

In total, we performed this model interpretability analysis on 1000 peptide pairs, which were randomly selected from the training and validation sets.

## Results and Discussion

### Intensity Ratio as a Stable Property to Distinguish Cross-Linked Peptide Pairs

Given a constant protein sequence, the probability of peptide bond fragmentation at specific sites remains fixed, regardless of the sample amount injected into the mass spectrometer. Therefore, spectra generated from the same linear peptide segment in different experiments are expected to have similar intensity distributions.^14,25^ This principle can be transferred to XL-MS, where spectra are generated from cross-linked peptides, which consist of two peptide segments. Regardless of the amount of sample introduced into the instrument, the absolute quantity ratio of these two peptides will always be 1:1, and the intensity ratio between the summed up fragment ion intensities orginated from the two linked peptides of a cross-link should remain constant. To test this hypothesis, we analyzed a large ground-truth data set^9^ (see Table 1 for an overview of all analyzed data sets), which includes 3 subdata sets. We selected cross-linked peptides that are represented by more than 30 CSMs. For each of these cross-linked peptides, we focused on the 15 CSMs containing the highest number of matched *b*- and *y*-fragments of both of the cross-linked peptides. Whereas different peptide pairs showed distinct intensity ratios as expected (standard deviation between groups = 0.674), the intensity ratios across most of CSMs of the same peptide pair varied within a narrow range (average standard deviation within groups = 0.097 after removing 5% outliers), effectively behaving as a constant (Figures 2A and S1). We also examined the stability of intensity ratios across different experimental replicates (Figure 2B). For CSMs with more than 15 repetitions in each experiment, we found that intensity ratios remained stable, showing no significant batch effects (Table S6).

**Figure 2:**
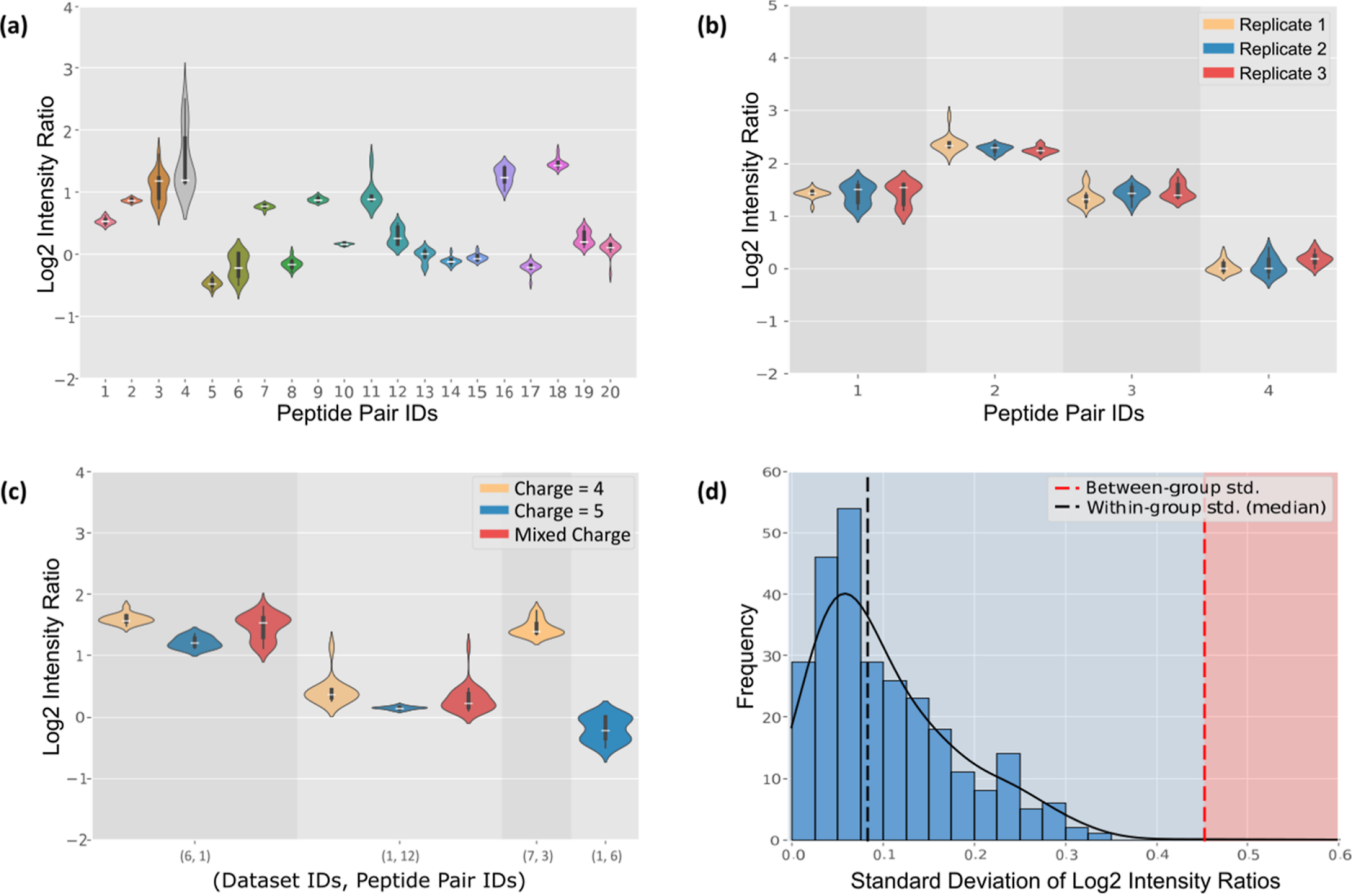
Analysis of intensity ratios in cross-link fragmentation. (a) Distribution of intensity ratios for 20 cross-links in from the ground-truth data set (first batch), each containing 15 highest-fragmentation-quality CSMs. All included CSMs feature both α and β peptides with modifications. The intensity ratios for 95% of the cross-links show an average std. of 0.097, significantly lower than the std. of 0.674 observed between different cross-links. The peptide pair (peptide ID 4) with a std. of 1.165 is an outlier and shown in gray color. Each peptide pair contains the CSMs with the same sequence and modification. The corresponding peptide sequences are shown in Table S2. The violin plot employs kernel density estimation (KDE) to depict the probability density of the data. Internal box plots display the interquartile range (IQR), with the median represented by a white line. Whiskers extend to the data range, excluding outliers. (b) Intensity ratio consistency of identical peptide pairs across three biological replicates, based on Mitochondria data sets. The corresponding peptide sequences are shown in Table S3. Violin plot settings are identical to (a). (c) Influence of precursor charge on bimodal distribution in intensity ratios in the ground-truth data set (first batch) and mitochondria data sets. The first number on the *x*-axis label indicates the data set ID (Table 1), and the second number represents the peptide pair’s ID (Tables S2 and S3); specific data sets and peptide sequences can be found in Tables 1 and S2 and S3, respectively. Violin plot settings are consistent with (a). (d) Comparison of intensity ratio standard deviations: within-group (same peptide pairs under different HCD energies) versus between-group (different peptide pairs) in the two-interactome data set. Between-group values are averaged across CSMs with identical peptide pairs but varying HCD energies.

In some peptide pairs, the distribution of intensity ratios exhibited a bimodal pattern. This is partially due to charge state-dependent differences, as shown by analyzing the top 15 CSMs corresponding to different precursor charge states (Figure 2C). However, we also found instances where CSMs of a single precursor charge state exhibited a bimodal distribution. Despite this, the overall fluctuation in intensity ratios was small and did not affect the ability to differentiate between CSMs based on intensity ratios. Curiously, we observed that some marginal outliers might result from mismatches caused by highly similar peptides. For example, in Figure S2A, the peptide pair with IDs 38 (GPGGSPGGLQKR-VTVKYDR) and 39 (GPGGSPGGLQKR-VTVKYDRR) differed only by a single R residue on the beta peptide. However, their intensity ratios showed significant divergence, and the outlier value of ID 39 was very close to the mean intensity ratio of ID 38. This suggests that the high similarity between these peptides may have led to partial mismatch of ID 39 spectra to ID 38 when relying solely on *m*/*z*-based matching scores. This case confirms that intensity-based features can help identify potential misassignments arising from *m*/*z*-based matching. Since collision energy is an important determinant of peptide fragmentation, we next analyzed the effect of varying HCD levels on the intensity ratios, using a published stepped HCD-MS2 data set (two-interactome data set).^16^ Within the HCD21–30 range, a considerable portion of peptide pairs indeed showed a monotonic change in intensity ratio as HCD increased (Figure S3). However, the standard deviation of intensity ratios within the same peptide pair was much smaller than the standard deviation between different peptide pairs. This suggests that, while HCD does impact the intensity ratios, minor changes in HCD energy do not result in significant changes. Our analysis of the two-interactome data set also suggests that the same CSMs are rarely detected across a range broader than HCD21–30 (Figure S4). Therefore, although larger HCD alterations may affect the intensity ratios in a more substantial manner, they will more likely change the set of detectable CSMs altogether.

### Intensity Ratio Model Building and Comparison

Having shown that intensity ratios can aid in distinguishing between different CSMs, it should be possible to differentiate true-positive from false positive CSMs by predicting the intensity ratio of a peptide pair and comparing it to the experimental intensity ratio obtained from the matched mass spectrum. To build a predictor for intensity ratios, we first analyzed which peptide pair features relate to experimental intensity ratios from the ground-truth data set (first batch). We first tested the correlation between intensity ratio and the length ratio of the two linked peptides and found the two values are positively correlated (Figure 3A,B). This is expected as longer peptide generates more fragments and thus higher summed intensity of fragment ions. However, fitting the intensity ratio based solely on length ratio showed limited correlation and a rather high mean absolute error (MAE). To better capture the complex features governing the intensity ratios, we employed machine learning techniques to design a predictive model.

**Figure 3:**
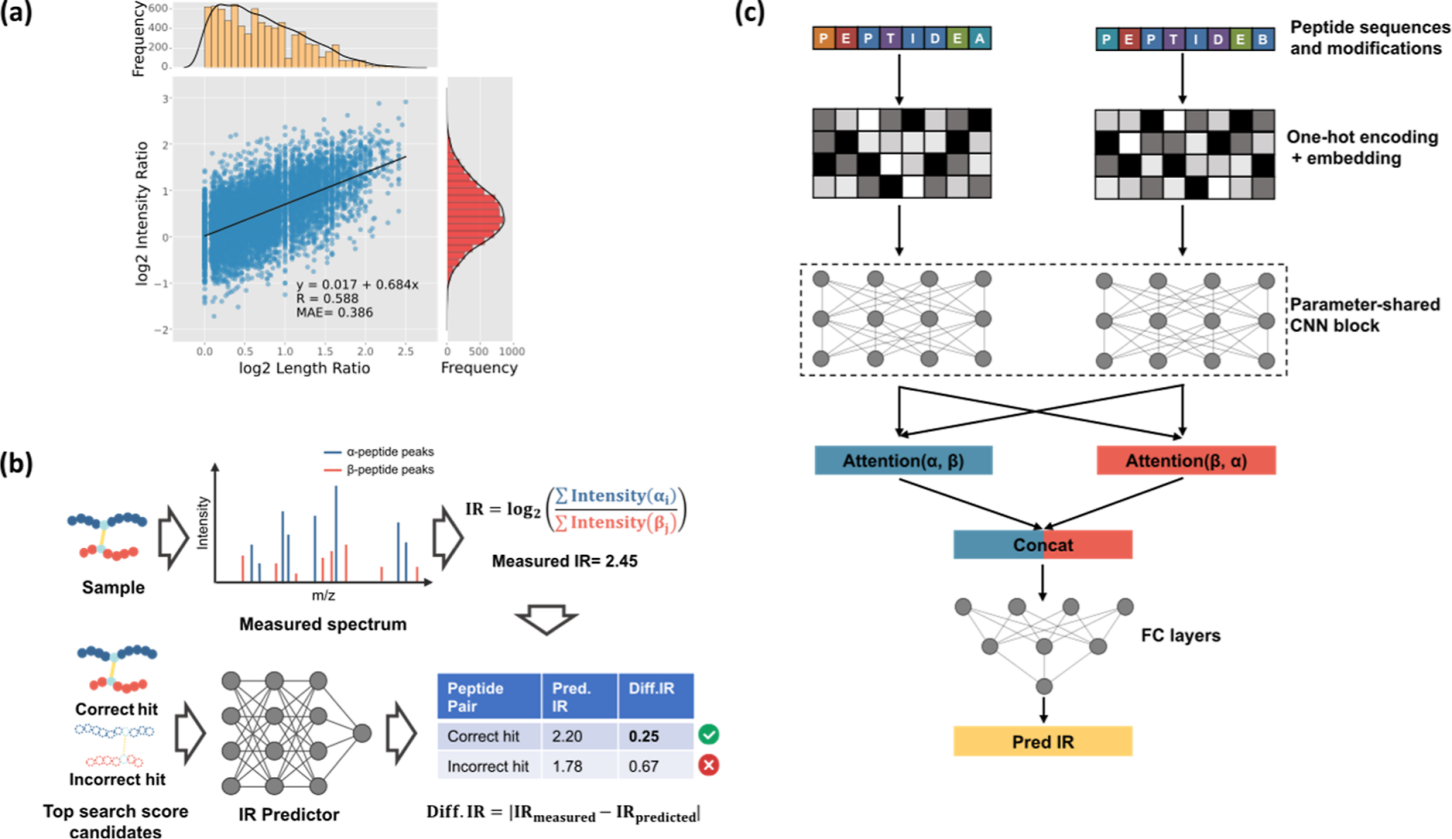
Rationale for predicting intensity ratios and overview of the AIRPred model architecture. (a) Correlation between the ratio of the length of the two linked peptides and the summed fragment intensity of the two linked peptides, analyzed using the Ground-truth data set (first batch). (b) Utilizing intensity ratios to differentiate between correct and incorrect hits. (c) Structure of the AIRPred model.

The architecture of our model, AIRPred, is shown in Figure 3c. AIRPred encodes the amino acid sequences and modifications of peptide pairs as input. We selected convolutional neural network (CNN) to extract information from the peptide sequences. The CNN’s convolutional filters are capable of capturing local neighborhood information, allowing the model to approximate the behavior of peptide fragmentation. Ablation experiments demonstrated that the CNN block is indeed the most critical component of the model—modifying this block resulted in a significant decrease in performance (Table 2). Additionally, because α-peptides and β-peptides in cross-linked peptides play equivalent roles, we employed a parameter-sharing strategy. This reduced the number of model parameters, making the model more robust and preventing overfitting, while also learning commonalities between peptides.

**Table 2:** Ablation Experiment Results

model	RMSE	MAE	Pearson Corr	Spearman Corr
AIRPred	0.206 ± 0.067	0.155 ± 0.050	0.937 ± 0.015	0.935 ± 0.014
w/o^a^ attention	0.244 ± 0.045	0.188 ± 0.034	0.912 ± 0.028	0.908 ± 0.028
w/o CNN	0.464 ± 0.027	0.366 ± 0.019	0.632 ± 0.038	0.616 ± 0.038
w/^b^ average pooling	0.258 ± 0.024	0.199 ± 0.019	0.905 ± 0.012	0.902 ± 0.013

a w/o: without using this method.

b w/: with using this method.

Following the CNN layers, we applied max pooling to extract information from different channels of the CNN output, and incorporated an attention layer to model the interactions between the α- and β-peptides. Ablation experiments showed that replacing max pooling with average pooling or removing the attention mechanism led to varying degrees of performance degradation (Table 2). This confirms the rationale behind our model.

### Model Performance Comparison

We benchmarked AIRPred against other common machine learning and deep learning models. We performed 5-fold cross-validation to select and compare the hyperparameters of each model (Figure 4A, detailed hyperparameters listed in Table S1). To simulate random mismatches (false hits) that occur during peptide identification, we randomly sampled values from the intensity ratio distribution in the training set and used these as prediction values.

**Figure 4:**
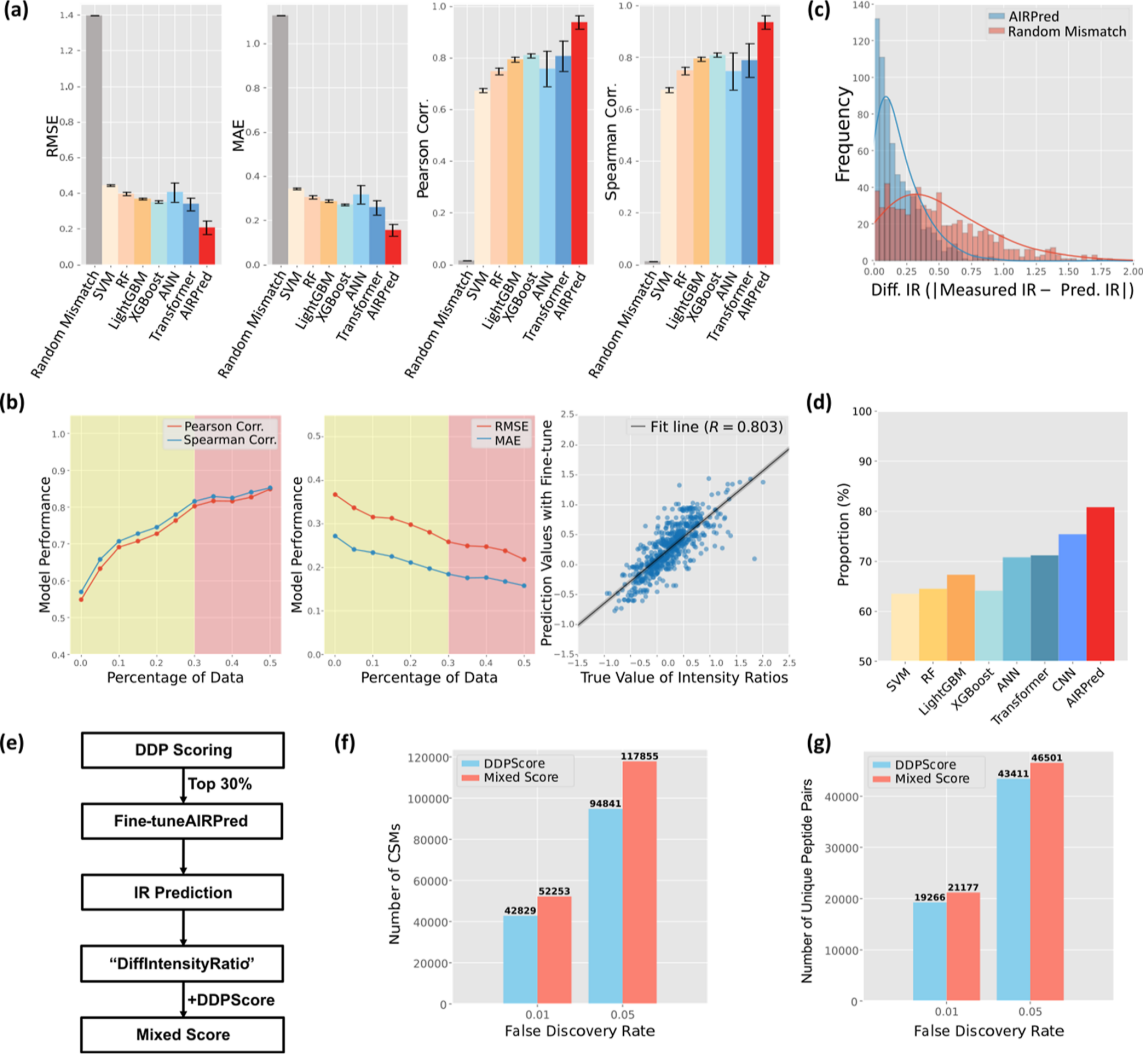
Validation and test results of AIRPred model. (a) 5-Fold cross-validation of AIRPred on the Ground-truth data sets, compared with other machine learning models. Error bars represent the standard error, while the top of each color bar indicates the mean metric value across the five folds. (b) Evaluation on an external data set to optimize to proportion of data used for fine-tuning. The borderline of yellow and red boxes (30%) is decided as the percentage of data for fine-turning. The AIRPred model was trained on ground-truth data sets (first, third and fourth batch) and tested on the two-interactome data set. (c) Comparison of AIRPred performance against random mismatch (simulated random incorrect matches) using difference in intensity ratio (Diff. IR) on the two-interactome data set, where Diff. IR = |(measured log_2_(IR)) – (predicted log­(IR))|. (d) Proportion of instances where models identify incorrect hits based solely on intensity information. Here, “better” indicates that model’s Diff. IR outperforms the random mismatch baseline. (e) Flowchart illustrating the fine-tuning process and mixed score calculation on the HEK293T data set. (f) Comparison of the number of identified CSMs using DDPScore and Mixed Score at different FDR levels on the HEK293T data set. (g) Same as (f), but restricted to **nonredundant peptide pairs**, showing unique cross-links identified at different FDR levels.

All machine learning models show better performance than a linear regression fit only with peptide length ratio, and AIRPred significantly outperformed other machine learning and deep learning models. Notably, the transformer model, which generally excels in sequence-based tasks,^26^ underperformed some traditional deep learning models. We believe this is due to the transformer’s large number of parameters and the relatively small size of the current training data set, which prohibits utilizing the transformer’s full potential.

We further evaluated the AIRPred’s performance on an external test set. The test set differed from the training set with regard to experimental conditions, particularly in species and HCD energy levels (Table 1). Despite these differences, the model predictions still showed moderate correlation with the actual values, with a MAE of 0.272 and a root-mean-square error (RMSE) of 0.367 (Figure 4B, middle panel).

To improve the usability of AIRPred under real experimental conditions, we fine-tuned the model on different proportions of the test data (Figure 4B). To prevent data leakage, we ensured that the peptide pairs used for fine-tuning did not appear in the remaining test set. With 30% of the test data used for fine-tuning, the Pearson and Spearman correlation coefficients both exceeded 0.8, and the MAE dropped below 0.2. Using more than 30% of data for fine-tuning resulted in only minimal performance gains. Because fine-tuning the model with a small amount of high-confidence CSM data is more feasible and relevant for practical applications, we chose the model fine-tuned with 30% of the data for further analysis.

As shown in Figure 4C,D, our model’s predictions were closer to the true intensity ratios than random predictions for more than 80% of individual CSMs. This demonstrates that the difference between predicted intensity ratio and measured intensity ratio, termed “DiffIntensityRatio”, is a viable candidate score to assist in CSM identification.

To evaluate the utility of DiffIntensityRatio in improving CSM identification under real experimental conditions and to assess its transferability to other cross-linkers, we tested AIRPred on the HEK293T data set (Figure 4E). By fine-tuning the model with the top 30% of CSMs and integrating DiffIRScore with DDPScore, we achieved both higher AUROC (area under the receiver operating characteristic curve) values and increased identification rates-detecting 22% more CSMs and 9.9% additional nonredundant cross-linked peptides at a 1% FDR threshold (Figures 4F,G, and S5). These results indicate that DiffIRScore captures information overlooked by DDPScore and can be effectively integrated into identification pipelines to enhance CSM discovery.

### Model Interpretation to Explore Amino Acids Contributions to Intensity Ratios

Although deep learning models are highly effective predictors, understanding the relationships they learn can be difficult. To enhance the interpretability of our model in the context of our experimental findings and to gain additional insights, we calculated shapley additive explanation (SHAP) values for each amino acid within every peptide sequence (Figure 5A). Compared to the SHAP base value, our model’s predictions are consistently closer to the true values obtained from experimental data, indicating that our model has indeed learned information related to amino acid sequences beyond just peptide length.

**Figure 5:**
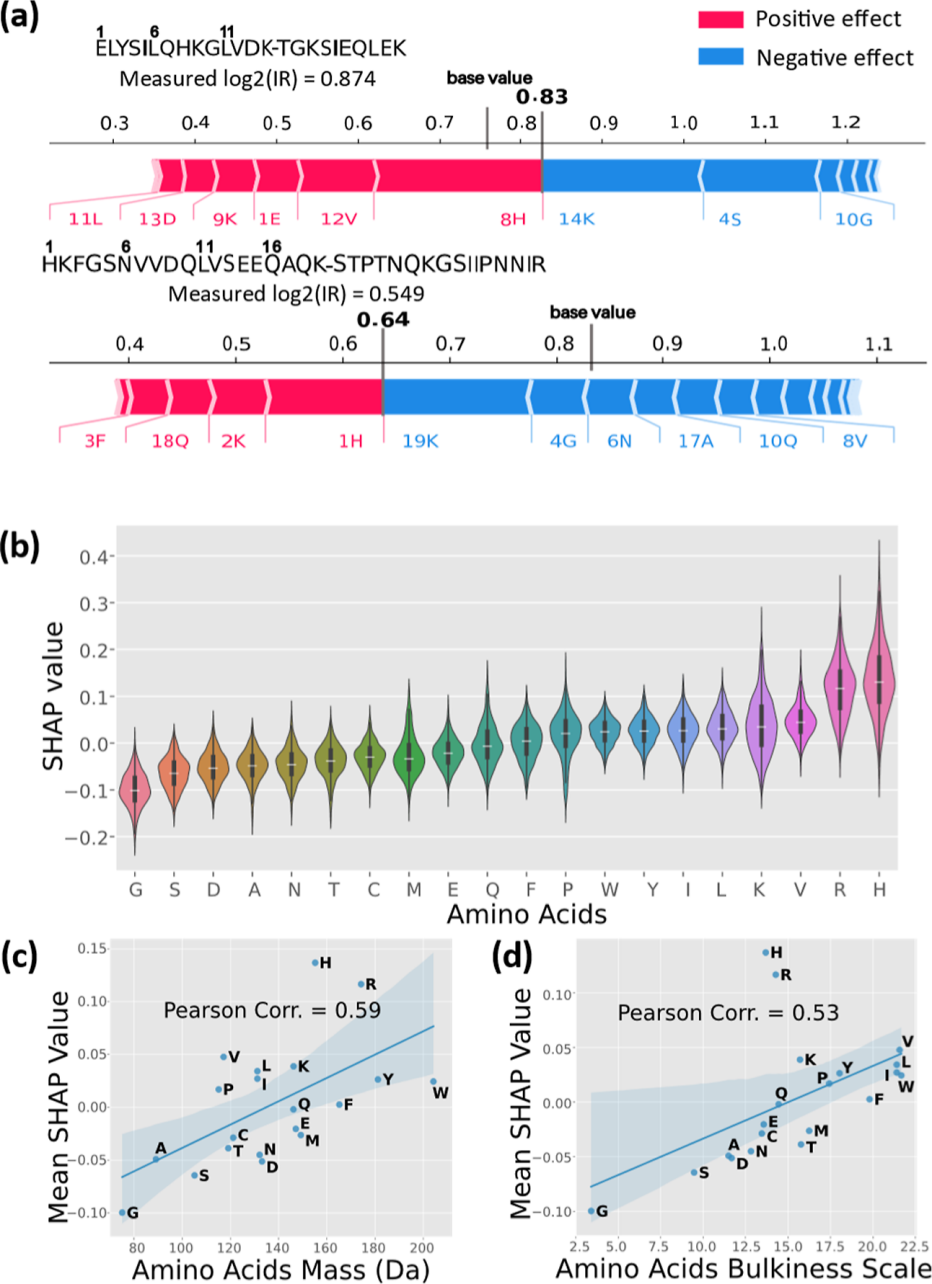
Interpretation analysis of the AIRPred model. (a) SHAP value attribution for peptide pairs. Example peptide pair sequences with SHAP attributions indicating the most influential amino acids within the sequence. The base value represents the average predicted intensity ratio by fixing the sequence of one of the linked peptides while randomly mutating each amino acid of the other peptide to another amino acid. The bold number is the predicted value for the original sequence of the cross-link. (b) Amino acid-specific SHAP value distributions, *n* = 1000 Peptide pairs in the ground-truth data sets. Violin plot settings are identical to Figure 1a. (c) Correlation between amino acid mass and mean SHAP value. (d) Correlation between amino acid bulkiness^35^ and mean SHAP value.

As illustrated in Figure 5A, the interpretability results reveal that even identical amino acids positioned differently within the same peptide sequence contribute variably to the final outcome. This finding aligns with our expectations, as the proximity of neighboring amino acids can alter the electron cloud density, thereby affecting the probability of peptide bond fragmentation,^27–29^ which in turn influences intensity. We aggregated the SHAP values for each amino acid across the entire test data set to explore the overall impact of different amino acid types on intensity (Figure 5B). We observed the most constant SHAP values for glycine and serine, whereas lysine, arginine, and histidine exhibited the highest variability (Figure 5B). Similar trends were found in a SHAP analysis by Meier et al.,^30^ which focused on the contribution of individual amino acids on peptide collision cross sections (CCS). The overlap between the SHAP trends from us and Meier et al. agree well with the known influence of CCS on MS signal intensity.^31^

Additionally, we found a correlation between the average SHAP values of amino acids and their masses and volumes, with Pearson correlation coefficients of 0.59 and 0.53, respectively (Figure 5C). This finding is consistent with previous research by Huang et al.,^32^ who used a knowledge-mining approach to demonstrate that larger steric hindrance in side chains can facilitate peptide fragmentation. Interestingly, the contributions of arginine and histidine to intensity were significantly greater than what would be expected based on their mass and volume rankings among amino acids. The comparison of cases ID38 and ID39 (Figure S2A) is consistent with this finding: although they differ only by an additional arginine residue on the β-peptide, ID39 exhibited a markedly lower intensity ratio than ID38. This discrepancy may be attributed to the basicity of histidine and arginine side chains, the protonation of which was shown to increase the susceptibility of peptide bonds to gas-phase cleavage.^33,34^

These results highlight that our deep learning model has effectively learned meaningful features, automatically extracting relevant physical quantities at the level of individual amino acids from the training data, despite only utilizing peptide pair sequences and modifications as input.

## Conclusions

Most current CSM scoring methods rely primarily on *m*/*z* information and overlook the importance of intensity data. We have shown that intensity ratios have the potential to serve as a new scoring metric to supplement existing scores for CSM identification. We have developed AIRPred to predict intensity ratios, and demonstrated that our model can enhance the CSM identification. These improvements were also observed on an XL-MS data set generated using DSBSO cross-linker, whereas AIRPred was trained and validated based on DSSO data. This suggests that our model is extendable to other cleavable cross-linkers.

We tested our model on an external test set and found that, even without using any data for training, the model’s predictions showed moderate correlation with the actual values. After fine-tuning with 30% of the data, the correlation between predicted and true values increased significantly. That means by leveraging high-confidence CSM identifications to adjust the model, we can optimize its ability to distinguish CSMs at different confidence levels, highlighting the potential of this approach as a robust CSM identification score. We also observed that the intensity ratio remained stable in the HCD range of 21–30. The effects of broader HCD variations on intensity ratios still need further experimental testing, but our initial analyses suggest that the same CSM is rarely detectable across HCD ranges wider than HCD21–30.

In the future, models succeeding AIRPred may take advantage of the fact that the intensity ratio of a peptide pair is likely to show a certain robustness against individual wrong CSMs, because it represents the mean value of a group of CSMs. This suggests that, for the training of next-generation models, we do not need to invest extensive time and resources in constructing ground-truth data sets; rather, we can select data from experiments and focus on peptide pairs with multiple CSMs. Furthermore, future models may take into account differences in intensity ratios for the same CSM across different experiments as a quality control metric, since we found that the intensity ratio is also related to fragmentation quality. Finally, new models and additional experimental data will be needed to enable the application of intensity ratios for the identification of noncleavable cross-links.

## Supplementary Material



## Data Availability

All mass spectrometry proteomics data were deposited to the ProteomeXchange Consortium via PRIDE, and the identifiers are provided in Table 2. The Scout software and user documentation are available at https://github.com/theliulab/Scout. The source code of AIRPred is available at https://github.com/zhangzh-orange/AIRPred.

## Notes

Model training procedures and hyperparameter optimization; benchmarking methods of baseline models (SVM, RF, LightGBM, XGBoost, ANN, transformer); details of the AIRPred architecture and feature encoding strategies; statistical analysis of intensity ratio reproducibility and batch effects; extended peptide pair annotations and modification-specific analyses; and supplementary figures on intensity ratio distributions and model performance (PDF)
