# Supplementary material for: AIRPred: A Deep Learning Model Predictor for Peptide Intensity Ratios in Cross-Linking Mass Spectrometry Improves Cross-Link Spectrum Matching
Source: Anal Chem. 2025 Aug 27;97(46):25500–9. doi: 10.1021/acs.analchem.5c03597 (PMC12658862; doi:10.1021/acs.analchem.5c03597)
Supplement: Supplementary file 1 [file ac5c03597_si_001.pdf]

AIRPred: A Deep Learning Model Predictor for Peptide Intensity Ratios in Cross-Linking Mass Spectrometry Improves Cross-Link Spectrum Matching

Zehong Zhang[1,3], Mei Wu[1], Max Ruwolt[1], Ying Zhu[1], Pin-Lian Jiang[1], Diogo Borges Lima[1], Fan Liu[*, 1,2]

1. Leibniz-Forschungsinstitut für Molekulare Pharmakologie (FMP), Berlin, 13125, Germany;
2. Charité Universitätsmedizin Berlin, 10117, Germany;
3. Freie Universität Berlin, Berlin, 14195, Germany

Contents:

1. Model Training and Baseline Models
2. Supplementary Materials for Intensity Ratio Property Analysis

1. Model Training and Baseline Models

Model Training and Hyperparameter Optimization

To ensure robust model performance and prevent overfitting, we employ a 5-fold cross-validation approach in training and validating all models, including the baseline models and our proposed model, AIRPred. In 5-fold cross-validation, the dataset is randomly partitioned into five equal subsets. Each subset serves as the validation set once while the remaining four subsets are used for training. This process is repeated five times, with each fold providing an evaluation metric that is averaged across folds, ensuring that the performance metrics are reliable and not skewed by any single data partition.

For hyperparameter optimization, we use random search, an efficient and scalable method that randomly samples hyperparameters from a predefined distribution. This approach allows us to explore a broad range of hyperparameter values without the computational expense of exhaustive grid search.

Baseline models

To evaluate the performance of our proposed model, we compare it with a series of widely used machine learning and deep learning baseline models: Support Vector Machine (SVM), Random Forest (RF), LightGBM, XGBoost, Artificial Neural Network (ANN), and Transformer. Each baseline model represents a distinct category of machine learning or deep learning approach, providing a comprehensive benchmarking framework. Below, we briefly describe each model and its relevance to our task.

Support Vector Machine (SVM)

The Support Vector Machine (SVM) is a powerful supervised learning algorithm commonly used for classification and regression tasks. SVM works by finding the optimal hyperplane that maximizes the margin between different classes in the feature space. For regression, we use Support Vector Regression (SVR), which extends the SVM principles to predict continuous values. SVM is particularly effective in handling high-dimensional spaces and is robust to overfitting, especially in cases with fewer outliers.

Random Forest (RF)

The Random Forest algorithm is an ensemble method that builds multiple decision trees during training and outputs the average prediction of individual trees for regression tasks or the majority vote for classification tasks. RF combines the advantages of decision trees with a bootstrapping approach, improving model stability and generalization by reducing overfitting. This model is particularly useful in capturing non-linear relationships and is effective on high-dimensional, unstructured data.

LightGBM

LightGBM, or Light Gradient Boosting Machine, is a gradient-boosting framework based on decision trees. It is designed to handle large datasets efficiently and provides fast training speeds through techniques such as histogram-based decision tree learning. By leveraging leaf-wise growth, LightGBM tends to have better accuracy and faster convergence than traditional gradient-boosting methods. This model is especially advantageous for large datasets and high-dimensional feature spaces.

XGBoost

XGBoost, or Extreme Gradient Boosting, is another popular gradient-boosting algorithm that builds an ensemble of weak learners in sequence, optimizing residual errors at each step. Known for its scalability, regularization, and ability to handle missing values, XGBoost has become a benchmark model for a wide range of machine learning tasks. Its objective function is specifically designed for regularization, making it robust to overfitting, especially in high-dimensional data scenarios.

Artificial Neural Network (ANN)

Artificial Neural Networks (ANNs) are deep learning models inspired by the structure of biological neural networks. ANNs consist of layers of interconnected nodes or neurons, each applying a non-linear transformation to its inputs. We use a fully connected ANN architecture with multiple hidden layers to capture complex non-linear relationships within the data. ANNs are particularly useful for high-dimensional data and are known for their ability to generalize on complex tasks, making them suitable for a wide range of predictive modeling problems.

Transformer

The Transformer model, originally developed for natural language processing tasks, has gained popularity in other fields due to its self-attention mechanism, which enables it to capture long-range dependencies within the input data. Unlike recurrent networks, Transformers process the entire input sequence simultaneously, allowing them to handle complex data structures efficiently. This architecture is particularly suited for sequence data and high-dimensional structured data and has demonstrated strong performance in predictive tasks involving time series, language, and structured input sequences.

Each baseline model was tuned for optimal performance based on 5-cross validation data, ensuring a fair comparison with our proposed approach. The diversity of these models allows for a robust assessment of model performance across different algorithmic paradigms, highlighting both linear and non-linear predictive capabilities.

Random mismatch

In addition, we randomly select intensity ratio values from the training set as to simulate random mismatches that occur during XL-MS peptide identification. This allows us to evaluate and compare our model’s ability to distinguish correct identifications from random mismatches.

AIRPred hyperparameters

Shown in Table S1.

Table S1. Hyperparameters of AIRPred model

Hyperparameter	Description
EPOCHS	150
BATCH_SIZE	32
Learning rate	5e-04
Loss function	Smooth L1 Loss
Optimizer	Adam
Learning rate scheduler	ExponentialLR
Learning rate decay factor	0.96
Dropout (attention layer)	0.3
Dropout (fully connected layer)	0.2

AIRPred Features

According to the default parameter settings in Scout, the maximum peptide search length is 60 amino acids. Therefore, the model's maximum single peptide segment length (either alpha-peptide or beta-peptide) is also set to 60. Peptides shorter than 60 amino acids are padded with zero vectors to reach this length. The input features consist of one-hot encoded vectors representing the types of amino acids. Each one-hot vector is 25-dimensional, encompassing 20 standard amino acids as well as the additional amino acids B (Asparagine or Aspartate), O (Pyrrolysine), U (Selenocysteine), X (Any / Unknown), and Z (Glutamine or Glutamate).

Loss function

To optimize our model's prediction accuracy, we incorporate the Smooth L1 Loss, a robust loss function commonly used in regression tasks. Unlike the Mean Squared Error (MSE) Loss, which is highly sensitive to outliers, Smooth L1 Loss provides a

balanced approach by combining properties of both L1 and L2 loss functions. This loss function is particularly effective in scenarios where outlier values can disrupt model training, as it reduces the influence of large residuals.

The Smooth L1 Loss is defined as follows:

$$L(x) = \begin{cases} 0.5x^2, & if\ |x| < 1 \\ |x| - 0.5, & otherwise \end{cases} \quad (1)$$

where $x = y_{pred} - y_{true}$ represents the difference between the predicted and true values.

This piecewise loss function operates as follows:

Quadratic Region: For small error values ($|x| < 1$), Smooth L1 Loss behaves similarly to L2 Loss, penalizing errors quadratically. This quadratic region encourages smaller adjustments when errors are minor, enabling smoother convergence.

Linear Region: For larger error values ($|x| \geq 1$), it transitions to a linear penalty, similar to L1 Loss, which reduces the impact of large errors or outliers. This linear region contributes to the model's robustness against outliers, preventing disproportionately large gradients.

By using Smooth L1 Loss, we aim to achieve stable model training with reduced sensitivity to outliers while maintaining the gradient smoothness necessary for effective optimization. In our experiments, this loss function demonstrated improved convergence stability and generalization compared to traditional MSE or L1 losses.

2. Supplementary Materials for Intensity Ratio Property Analysis

Table S2. Sequence Annotations of Peptide Pairs Corresponding to Figures 2a and S1

id	Alpha peptide	Beta peptide	Alpha modification(s)	Beta modification(s)	Duplicates
1	EIVSGMKYIQHTYR	IDKTDYMVGSYGPR	M6 (Oxidation of Methionine)	M7 (Oxidation of Methionine)	113
2	LETHEEASVKMLPTYVR	MQKEMDR	M11 (Oxidation of Methionine)	M1 (Oxidation of Methionine); M5 (Oxidation of Methionine)	97
3	VIPSFMCQAGDFTNHNGTGGKSIYGSR	ALCTGEKGFGYK	C7 (Carbamidomethyl); M6 (Oxidation of Methionine)	C3 (Carbamidomethyl)	81
4	IMGFHRPGSGAEEESQTKSK	KLLMNSEQR	M2 (Oxidation of Methionine)	M4 (Oxidation of Methionine)	75
5	ISFNDFLAVMTQKMSEK	KMISEVDREGTGK	M10 (Oxidation of Methionine); M14 (Oxidation of Methionine)	M2 (Oxidation of Methionine)	57
6	ELKVAMR	KEEMKK	M6 (Oxidation of Methionine)	M4 (Oxidation of Methionine)	53
7	LETHEEASVKMLPTYVR	MQKEMDR	M11 (Oxidation of Methionine)	M5 (Oxidation of Methionine)	52
8	EILLAMLMVDKEK	KGYVMASDLR	M6 (Oxidation of Methionine); M8 (Oxidation of Methionine)	M5 (Oxidation of Methionine)	52
9	MESMAVATDGGERPGVPAGSGLSASQR	KLLMNSEQR	M1 (Oxidation of Methionine); M4 (Oxidation of Methionine)	M4 (Oxidation of Methionine)	51
10	ALYQDETGTMKTSEEDSR	FSDLTKSLQECR	M10 (Oxidation of Methionine)	C11 (Carbamidomethyl)	46
11	TVIGCSGFHGDCLTLTKIIEAR	DSPKCYK	C5 (Carbamidomethyl); C12 (Carbamidomethyl)	C5 (Carbamidomethyl)	44
12	LETHEEASVKMLPTYVR	LIGGKYMGELVR	M11 (Oxidation of Methionine)	M7 (Oxidation of Methionine)	44
13	ECFSLYDKQQR	IKATDLMVAMR	C2 (Carbamidomethyl)	M7 (Oxidation of Methionine); M10 (Oxidation of Methionine)	42
14	ENFPNFLSACDKK	IEKPSLLTMMK	C10 (Carbamidomethyl)	M9 (Oxidation of Methionine); M10 (Oxidation of Methionine)	41
15	GILAADESVGSMAKR	CVLKISER	M12 (Oxidation of Methionine)	C1 (Carbamidomethyl)	39
16	IMGFHRPGSGAEEESQTKSK	FEEAMKLR	M2 (Oxidation of Methionine)	M5 (Oxidation of Methionine)	37
17	ECFSLYDKQQR	IKATDLMVAMR	C2 (Carbamidomethyl)	M10 (Oxidation of Methionine)	34
18	SIIGMIDMFHKYTGR	MSNTQAER	M5 (Oxidation of Methionine); M8 (Oxidation of Methionine)	M1 (Oxidation of Methionine)	33
19	KHNLCGETEEEK	ISAYMKSSR	C5 (Carbamidomethyl)	M5 (Oxidation of Methionine)	31
20	KLLMNSEQR	FEEAMKLR	M4 (Oxidation of Methionine)	M5 (Oxidation of Methionine)	30

Note: For the UnsameMod analysis in Figure S1, no controls were applied to ensure that alpha and beta modifications remained consistent. All CSM data used here is derived from the Ground-truth dataset (first batch), identified as Dataset ID 1.

Table S3. Sequence Annotations of Peptide Pairs Corresponding to Figure 2b

Peptide Pair IDs	Alpha peptide	Beta peptide	Alpha modification(s)	Beta modification(s)	Duplicates (replicate 1)	Duplicates (replicate 2)	Duplicates (replicate 3)
1	TPFLLSGTSYKDLMPHDLAR	LMLDLNKAK	M14 (Oxidation of Methionine)	M2 (Oxidation of Methionine)	18	27	23
2	THTQDAVPLTLGQEFSGYVQQVKYAMTR	IKAAMPR	M26 (Oxidation of Methionine)	M5 (Oxidation of Methionine)	27	24	11
3	DGKTLNDELEIIEGMK	DDAMLLKGK	M15 (Oxidation of Methionine)	M4 (Oxidation of Methionine)	23	23	19
4	IVQAEGEAEAAKMLGEALSK	MLGEALSKNPGYIK	M13 (Oxidation of Methionine)	M1 (Oxidation of Methionine)	19	20	44

Note: Duplicates (replicate 1) indicates the number of times the peptide pair is repeated in Dataset ID = 5, specifically in the Mitochondria dataset (replicate 1). Similarly, Duplicates (replicate 2) and Duplicates (replicate 3) represent the number of times the peptide pair's CSMs is repeated in the Mitochondria dataset (replicate 2) and Mitochondria dataset (replicate 3), respectively.

Table S4. Sequence Annotations of Peptide Pairs Corresponding to Figures S2a (without Any Modifications).

id	Alpha peptide	Beta peptide	Alpha modification(s)	Beta modification(s)	Duplicates
21	SIQEIQELDKDDESLRK	YKEALLGR			229
22	ASVFVKLQK	LAILYAKR			175
23	IELGDVTPHNIKQLK	FYKDVLEVGELAK			160
24	ENITEDEAKEVHR	SLKIAAGIFK			153
25	EVDDLFREADIEPNGKVK	KGYVMASDLR			132
26	KYEVLALLYHEPPEDDK	VINVNAKNYK			107
27	ELQAAEYLEKHQIK	PKEYLISLLER			105
28	GKNYQNGDVFGDEYR	NQENLEKSASSNVR			94
29	KVAAIEALNDGELQK	LAILYAKR			88
30	DVAKHLGFQSAEEALR	YPKEDSEIR			86
31	DQVKQQYEHSVQISR	GLLFSASKQDLLR			82
32	GKNYQNGDVFGDEYR	IKPVEEVK			79
33	GSGSSKPTGSSR	LEAGGKGTER			75
34	DQVKQQYEHSVQISR	KVNFTVEVLLPDK			74
35	IAEKIDEFLATGK	NVSQAIHKYNAYR			71
36	ELYSILQHKGLVDK	TGKSIEQLEK			65
37	VYDLTKFLEEHPGGEEVLR	AEQSDEAVKYYTLEEIQK			64
38	GPGGSPGGLQKR	VTVKYDR			59
39	GPGGSPGGLQKR	VTVKYDRR			59
40	YDEFIHKITLPGR	EADIEPNGKVK			58

Table S5. Sequence Annotations of Peptide Pairs Corresponding to Figures S2b (with Modification on One Peptide Only).

id	Alpha peptide	Beta peptide	Alpha modification(s)	Beta modification(s)	Duplicates
41	EVDDLFREADIEPNGKVK	KGYVMASDLR		M5 (Oxidation of Methionine)	287
42	LFHPKLIIAGTSCYSR	GVKSVDPK	C13 (Carbamidomethyl)		123
43	DLNHVCVISETGKAK	VASLLVKR	C6 (Carbamidomethyl)		121
44	SIIGMIDMFHKYTGR	DGKIEK	M5 (Oxidation of Methionine); M8 (Oxidation of Methionine)		113
45	ISFNDFLAVMTQKMSEK	DTKEEILK	M10 (Oxidation of Methionine); M14 (Oxidation of Methionine)		108
46	KQLISEKPSQEDGNTTEEFDSFR	FEEAMKLR		M5 (Oxidation of Methionine)	102
47	EADIEPNGKVK	KGYVMASDLR		M5 (Oxidation of Methionine)	98
48	TIYFFGDKTMPGGNDHEIFTDPR	IEFYELDKK	M10 (Oxidation of Methionine)		90
49	SIIGMIDMFHKYTGR	DKNEDK	M5 (Oxidation of Methionine); M8 (Oxidation of Methionine)		86
50	NKPGEIAKYMETVK	DILLQGLKAIGSK	M10 (Oxidation of Methionine)		82
51	VYDLTKFLEEHPGGEEVLR	EMSKTFIIGELHPDDRPK		M2 (Oxidation of Methionine)	74
52	VEQILAEFQLQEEDLKK	MQKEMDR		M1 (Oxidation of Methionine); M5 (Oxidation of Methionine)	73
53	EALKTLGLCTEDEDLQDDGHK	ITLDKFK	C9 (Carbamidomethyl)		70
54	ISFNDFLAVMTQKMSEK	DTKEEILK	M14 (Oxidation of Methionine)		68
55	EILLAMLMVDKEK	SKLTSLGEK	M6 (Oxidation of Methionine); M8 (Oxidation of Methionine)		68
56	IVKPNNNNMPSSDDGLEHNK	IQNGKAPR	M9 (Oxidation of Methionine)		65
57	NLEAQHKELEEK	HEQMKK		M4 (Oxidation of Methionine)	65
58	ITVGVDGSVYKLHPSFK	LIGGKYMGELVR		M7 (Oxidation of Methionine)	64
59	YASICQQNGIVPIVEPEILPDGDHDLKR	PHSYPALSAEQKK	C5 (Carbamidomethyl)		64
60	IMGFHRPGSGAEEESQTKSK	QQDSDKLNSLSVPSVSK	M2 (Oxidation of Methionine)		63

Statistical analyses revealed consistent IR (intensity ratio) values across experiments, with no substantial batch effects. As summarized in Table S3, we evaluated data normality using the Shapiro-Wilk test, which indicated non-normal distributions for most groups (e.g., Replicates 1 and 3 of Peptide Pair 1; Replicate 1 of Peptide Pair 3; p-values < 0.05). Levene's test for homogeneity of variance showed p-values > 0.05 for all peptide pairs, suggesting no significant variance heterogeneity. Due to these deviations from normality, non-parametric methods (Kruskal-Wallis test) were employed to assess potential batch effects, ensuring robust statistical inference.

The Kruskal-Wallis test revealed a marginal batch effect in Peptide Pair 3 ($p = 0.04081$), though pairwise Wilcoxon tests did not reach strict significance (all $p > 0.05$). Additionally, low coefficients of variation (CV < 11% across all replicates) further supported experimental reproducibility, confirming the absence of substantial batch effects.

Table S6. Statistical Summary Table of Batch Effect Evaluation Corresponding to Figure 2b

Peptide Pair IDs	1			2			3			4		
Replicates	1	2	3	1	2	3	1	2	3	1	2	3
Shapiro-Wilk normality test (p value)	0.004	0.406	0.002	0.348	2.55e-05	0.164	0.020	0.147	0.128	0.117	0.554	0.079
Levene's test (p value)	0.098			0.518			0.631			0.433		
Kruskal-Wallis Test: χ^2	1.89			1.88			6.40			5.65		
Kruskal-Wallis Test: p-value	0.389			0.391			0.041			0.059		
Replicate Pairs	1 vs. 2	1 vs. 3	2 vs. 3	1 vs. 2	1 vs. 3	2 vs. 3	1 vs. 2	1 vs. 3	2 vs. 3	1 vs. 2	1 vs. 3	2 vs. 3
Pairwise-Wilcox Test (p value)	0.57	0.58	0.58	0.97	0.46	0.46	0.079	0.056	0.796	1.000	0.086	0.095
CV (%)	3.20			4.15			5.90			10.50		

Statistical analyses demonstrated that HCD energy levels (21–30) had no significant impact on IR values. The data significantly deviated from normality (Shapiro-Wilk p-value < 2e-16) and exhibited heterogeneous variance across HCD energy levels (Levene's test, p-value = 0.0014), justifying nonparametric analyses.

The Kruskal-Wallis test revealed an extremely significant effect of peptide sequence on IR values ($\chi^2 = 589.38$, p-value < 2.2e-16), indicating that peptide identity was the dominant source of variation. In contrast, the effect of HCD energy was marginally non-significant ($\chi^2 = 7.713$, p-value = 0.052) with a negligible effect size ($\eta^2 = 0.00564$, "small" magnitude). These results indicate that while peptide identity critically influences IR values, HCD energy variations contribute minimally and can likely be omitted in future experimental designs.

Table S7. Statistical Summary Table of HCD Effect Corresponding to Figure 2d

Test	Statistic / Value	p-value	Effect Size (η^2)	Magnitude
Shapiro-Wilk normality test	W = 0.913	< 2e-16	-	-
Levene's test (homogeneity of variance)	F = 5.23	0.0014	-	-
Kruskal-Wallis (Peptide effect)	$\chi^2 = 589.38$, df = 208	< 2.2e-16	0.604	Large
Kruskal-Wallis (HCD energy effect)	$\chi^2 = 7.71$, df = 3	0.052	0.006	Small

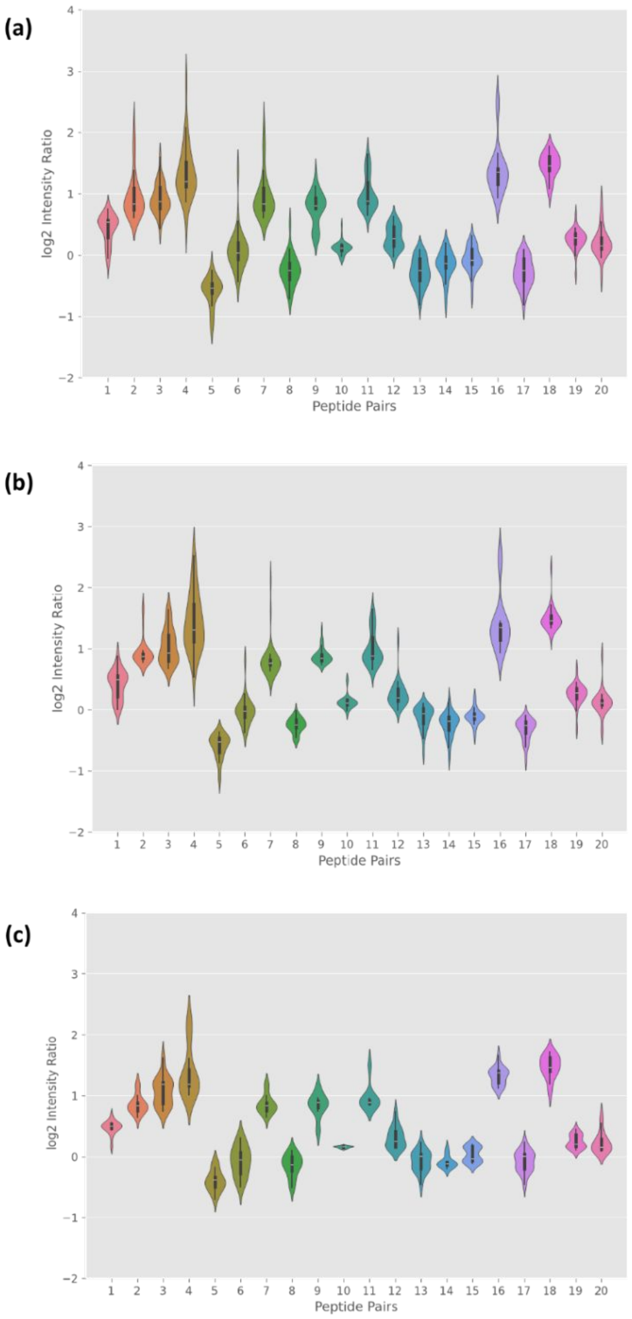

Figure S1. Distribution of intensity ratios for 20 cross-links in from the Ground-truth dataset (first batch). (a) For each peptide pair, a random selection of 30 CSMs is used to display the distribution of intensity ratios, allowing identical peptide sequences with different modifications. The violin plot employs kernel density estimation (KDE) to depict the probability density of the data. Internal box plots display the interquartile range (IQR), with the median represented by a white line. Whiskers extend to the data range, excluding outliers. (b) For each peptide pair, a random selection of 30 CSMs is used to display the distribution of intensity ratios, restricting identical peptide sequences to the same modification. Violin plot settings are identical to (a). (c) For each peptide pair, the top 15 highest-quality CSMs are selected to display the distribution of intensity ratios, allowing identical peptide sequences with different modifications. Violin plot settings are identical to (a).

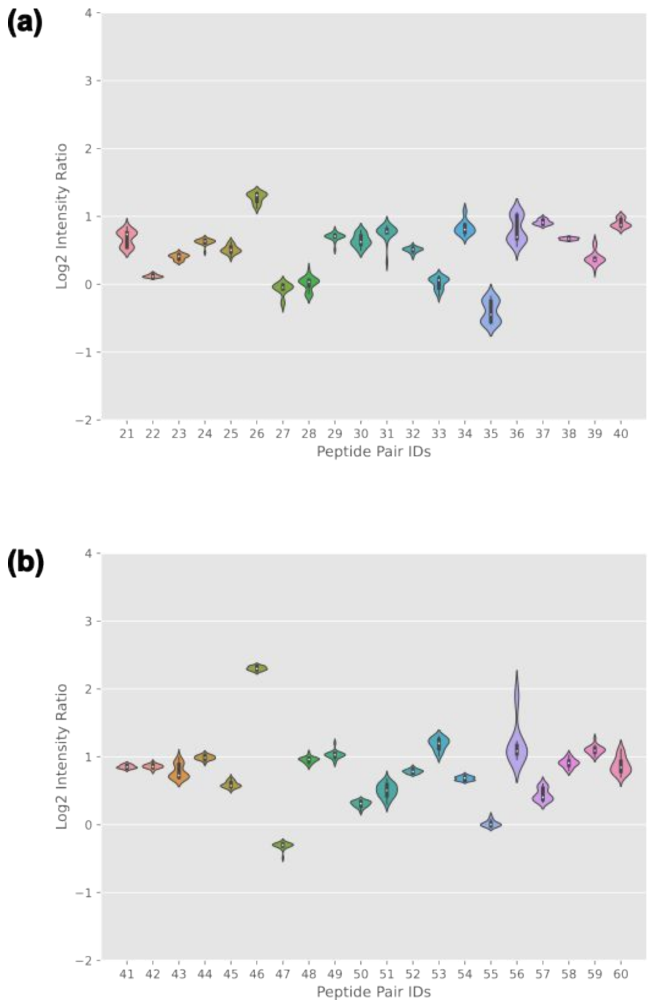

Figure S2. Distribution of intensity ratios for 20 cross-links in from the Ground-truth dataset (first batch). (a) Distribution of intensity ratios for 20 cross-links in from the Ground-truth dataset (first batch), each containing 15 highest-fragmentation-quality CSMs. All included CSMs feature both α and β peptides without modifications. (b) Distribution of intensity ratios for 20 cross-links in from the Ground-truth dataset (first batch), each containing 15 highest-fragmentation-quality CSMs. All included CSMs feature only one modified peptide (either α or β).

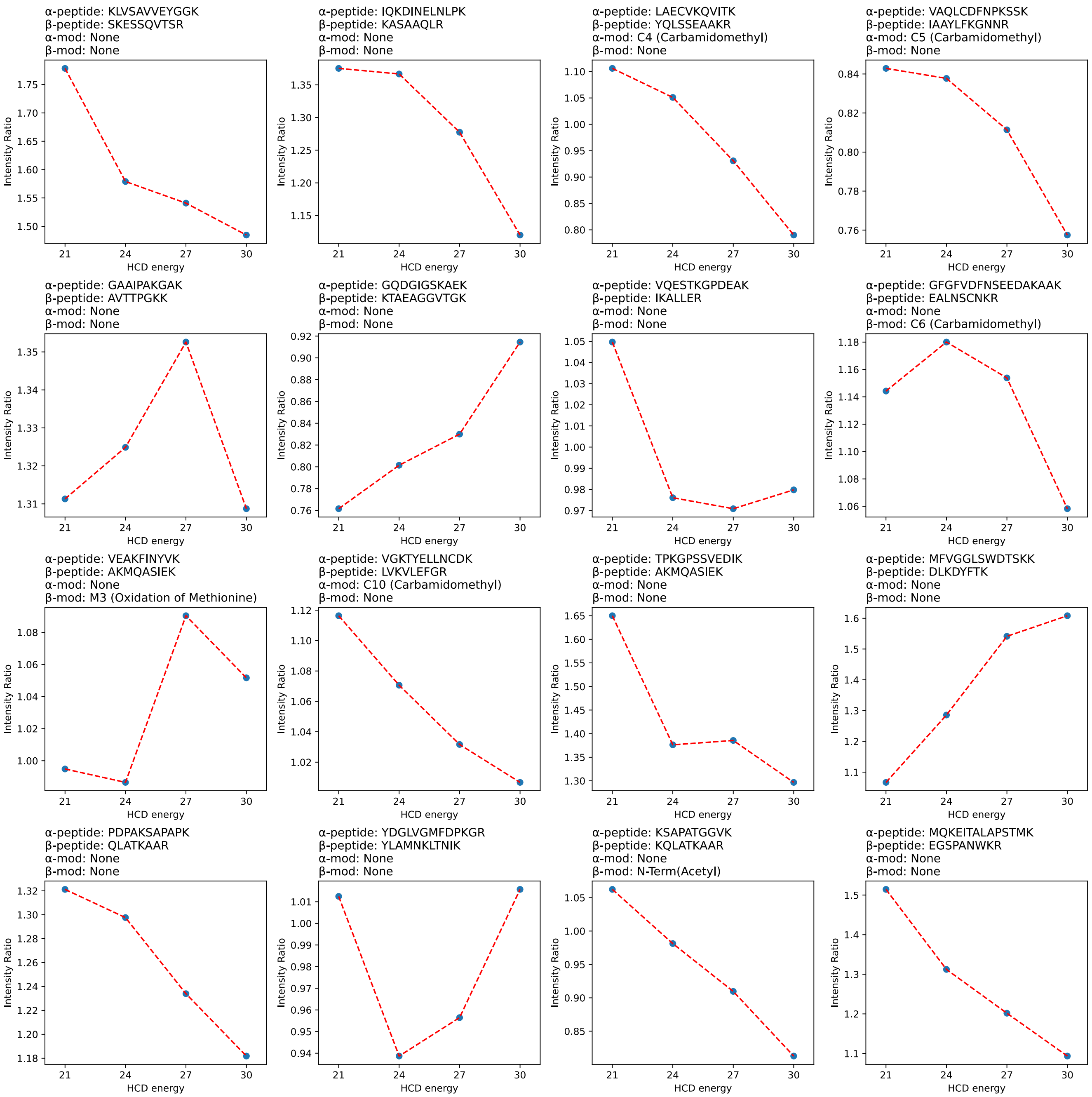

Figure S3. Variation in intensity ratios of randomly selected CSMs with changes in HCD in the Two-interactome dataset.

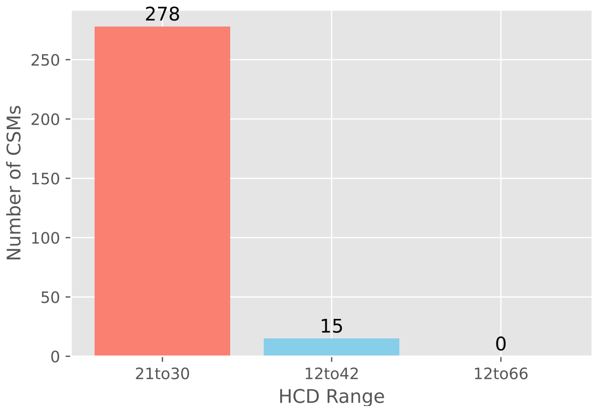

Figure S4. Number of unique CSMs across different HCD energy ranges in the Two-interactome dataset. The CSM count represents the number of unique CSMs detected at every measured HCD energy point within a specified range. For example, 278 CSMs were consistently identified at all measured HCD energy points within the 21-to-30 range (i.e., at 21, 24, 27, and 30).

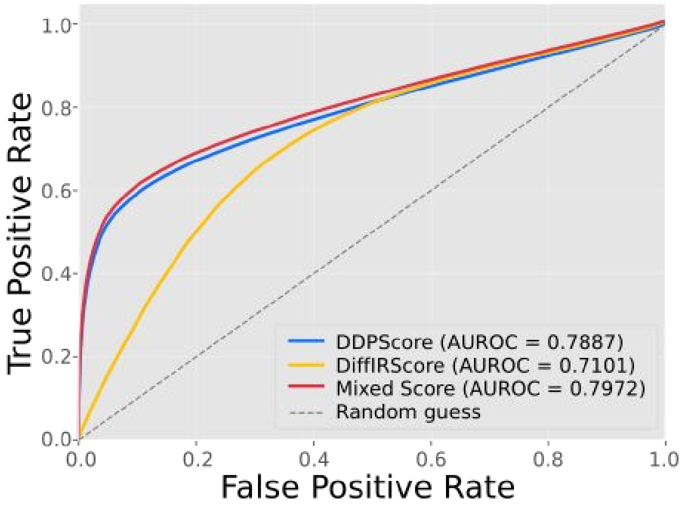

Figure S5. ROC curves for DDPScore and Mixed Score on the HEK293T dataset.
